# Bone augmentation in dental implants among Indian patients

**DOI:** 10.6026/973206300191307

**Published:** 2023-12-31

**Authors:** Akshayraj Langaliya, Pravin Uttam Gaikwad, Tarun Kumar Singh, Washim Akram, Vineet Nair, Prajakta Barapatre, Hitika P Doda, Dhaval Niranjan Mehta

**Affiliations:** 1 Department of Conservative Dentistry and Endodontics, AMC Dental College and Hospital, Gujarat, India; 2 Department of Prosthodontics Crown and Bridge, M.A. Rangoonwala College of dental sciences and research Centre, Pune, India; 3 Department of Dentistry, AIIMS, Bathinda, Punjab, India; 4 Department of Dentistry, Raiganj Medical College and Hospital, West Bengal, India; 5 Department of Periodontology, Dr. R Ahmed Dental College and Hospital, Kolkata, West Bengal, India; 6 Department of Prosthodontics Crown and Bridge, Mahatma Gandhi Dental College & Hospital, Jaipur, India; 7 Midtown Dental GroupUnited States; 8 Department of Oral Medicine and Radiology, Narsinbhai Patel Dental college and Hospital, Sankalchand Patel University, Visnagar, Gujarat, India

**Keywords:** Bone augmentation, dental implants, xeno-graphic titanium mess, e-PTTE, autologous bone grafts

## Abstract

It is of interest to compare guided bone regeneration (GBR) with titanium mesh, alveolar distraction osteo-genesis (DO), GBR with auto-genous bone and e-PTFE membranes and onlay bone grafts. This helps to restore vertically lacking alveolar ridges and their capacity to sustain the vertical bone growth acquired both prior to and following implant placement. The parameters such as (i) success and survival of dental implants (ii) peri-implant clinical parameters after prosthetic loading at 1 year, 2 year and 3 year follow up (iii) resorption of alveolar ridge regenerated before placement of dental implants and after placement of dental implants were assessed. Data shows that the distraction osteo-genesis is more predictable for long-term prognosis of vertical bone growth. However, all methods help to repair the vertically resorbed edentulous ridges.

## Background:

In implant surgery, implant location, fundamental stability, soft tissue shape recuperation, and other crucial elements linked to a proper implantation rehabilitation are influenced by the volume and nature of alveolar bone in the area of dental implant [1-2]. Following the loss of teeth, the alveolar bone typically experiences secondary resorption and degeneration, resulting in a steady reduction in the height as well as width of the alveolar ridge that is no longer adequate for placement of dental implants [3- 4]. As a result, a crucial aspect in implant dentistry is the alveolar bone reconstruction around the dental implant. Alveolar bone defects can be recovered using a variety of clinical techniques, such as distraction osteogenesis, bone extrusion, guided bone regeneration (GBR), on lay bone transplantation, and bone splitting technique [5- 6].

The absence of a firm foundation for the bone transplants in the region of defect could cause them to shift due to local stress, which would cause the bone-augmented area to collapse and not produce the desired result [7- 8]. Thus, it is essential for the barrier membranes in GBR approach to have enough retention potential, supportability and stiffness in order to ensure high biocompatibility [9- 10]. Conventional barrier membranes, on the other hand, are somewhat flexible and struggle to provide sufficient retention and safeguarding for the bone regeneration areas, despite having the ability to isolate cells selectively [11-12]. Examples of these membranes include nonabsorbable expanded poly-tetra-fluor-ethylene membranes (ePTFE) and absorbable collagen [13- 14].

It is challenging to maintain an appropriate and stable space for bone regeneration when typical barrier membranes are placed to big bone defects due to their stiffness, and it is simple to cause micromotion that compromises blood flow [15- 16]. Several clinical studies indicate that titanium mesh has excellent mechanical qualities and remarkable osteogenic performance upon application when the alveolar bone has extensive vertical bone defects or horizontal bone defects [17-18]. A technique for repairing vertically degenerated alveolar ridges is alveolar DO. It was first used to treat orthopaedic conditions, but in recent years, it has also been used to treat maxillofacial abnormalities. Correcting vertical deficiencies of the edentulous alveolar ridges has been proposed [19- 20].

Although the published data are hard to compare because multiple methodologies are utilised to assess implant persistence and achievement rates, in above mentioned techniques for ridge augmentation [21-22]. Therefore, there is still considerable debate regarding which of these different methods for bone augmentation in resorbed alveolar ridge is more trustworthy, even though there have been a sizable number of papers on the subject. To the best of the author's understanding, no comparative research comparing these approaches has been carried out before [23- 24]. Therefore, it is of interest to compare guided bone regeneration (GBR) with Titanium mesh, alveolar distraction osteogenesis (DO), GBR with autogenous bone and e-PTFE membranes and onlay bone grafts for their capacity to restore vertically lacking alveolar ridges and their capacity to sustain the vertical bone growth acquired both prior to and following implant placement.

## Methods and Materials:

89 patients were included in the study where 22 patients (34 implants) were managed through GBR with xenographic titanium mesh (category 1), 22 patients (35 implants) were managed with GBR with e-PTTE membrane and autogenous bone (category 2), 23 (34 implants) patients were managed with alveolar DO (category 3). 20 Patients were managed with onlay bone grafts (category 4). All patients were rehabilitated with implant supported prostheses after three to five months. There was assessment of these parameters (i) success and survival of dental implants (ii) peri implant clinical parameters after prosthetic loading at 1 year,2 year and 3 year follow up (iii) resorption of alveolar ridge regenerated before placement of dental implants and after placement of dental implants.

Over a duration of three years, patients in systemically good health who had imperfections in the vertical alveolar ridge were chosen for surgical treatment of the deficiency in order to enhance crown-to-implant ratio, implant support, the, and the aesthetics of implant-borne prostheses made in the edentulous areas.

The following conditions applied to patients' exclusion:

[1] Vertical edentulous ridge defects linked to a pronounced knife-edge ridge

[2] Bone abnormalities after tumour excision

[3] Abusing tobacco (smoking over 15 cigarettes daily)

[4] Severe illness of the liver and kidneys

[5] Radiation history in the head & neck area

[6] Uncontrolled diabetes

[7] Chemotherapy for management of malignant tumours at the time of surgery

[8] A periodontal disease that is actively affecting the remaining teeth

[9] Mucosal disorders in the areas that need to be addressed, like lichen planus

[10] Inadequate dental care

[11] Patients who do not comply

## Statistical analysis

The Levene test was used to assess homogeneity of variance. The Kruskal-Wallis ANOVA exact analysis with the use of the Monte Carlo technique to determine probability was utilised for multiple comparisons because the Levene test was significant. The Mann-Whitney U-exact test was utilised for multiple sample assessments.

## Results:

The bone resorption was low in DO and onlay bone grafts while it was high in GTR ± ePTTE and GTR± Titanium mesh. Current data is statistically significant (p<0.001). The values of bone resorption were comparable in GTR ± ePTTE and GTR± Titanium mesh with no statistically distinct variation. The values of bone resorption were comparable in DO and onlay bone grafts with no statistically distinct variation (Table 1).

The cumulative survival rate in GBR ± Titanium mesh membranes during 1-2 year follow up was 100%, while the Cumulative success rate during 1-2 year follow up was 84.4%. The cumulative survival rate in GBR ± ePTTE membranes during 2-3 year follow up was 100%, while the cumulative success rate during 2-3 year follow up was 74%. The cumulative survival rate in onlay bone grafts during 2-3 year follow up was 100%, while the Cumulative success rate during 2-3 year follow up was 95.2% (Table 2).

## Discussion:

Alveolar bone defects can be recovered using a variety of clinical techniques, such as distraction osteogenesis, bone extrusion, guided bone regeneration (GBR), onlay bone transplantation, and bone splitting technique [15- 16]. The published results, however, are difficult to compare because different procedures are used to evaluate implant persistence and accomplishment rates in the ridge augmentation techniques discussed above [12- 13]. Consequently, despite a large number of articles on the topic, there is still much disagreement about which of these several techniques for bone augmentation in resorbed alveolar ridge is more reliable [14,17].

In comparison to implants inserted into native, non-regenerated bone, larger amounts of peri-implant loss of bone may occur following the removal of the barrier membrane in GBR technique. Additionally, the proportion of success of dental implants inserted in regions subjected to vertical GBR treatment are substantially lower than those of implants inserted in native, unreconstructed bone [25-26]. Within the parameters suggested elsewhere [27] bone resorption readings have been documented in these papers, and cumulative success ranged from eighty-nine percent to 99.99% following follow-up intervals spanning between 3 years and 15 years [23-24].

The volume and type of alveolar bone around a dental implant determine implant position, basic stability, soft tissue shape recovery, and other important factors related to successful implantation rehabilitation [18-19]. After teeth are lost, the alveolar bone usually undergoes secondary resorption and degeneration. This causes the alveolar ridge's height and width to gradually decrease to the point where it is no longer suitable for the implantation of dental implants. As such, the alveolar bone repair surrounding the dental implant is an essential component of implant dentistry [20- 21]. Numerous clinical procedures, including distraction osteogenesis, bone extrusion, guided bone regeneration (GBR), onlay bone transplantation, and bone splitting technique, can be used to restore alveolar bone abnormalities [23,24].

Alveolar bone resorption levels one to three years following the beginning of prosthetic loading fell between the ranges suggested elsewhere [27] and matched the outcomes of implants inserted into native bone [25-26]. A 100% probability of survival and a 95.2 percent rate of success appear to support the idea that implants inserted into neo-generated bone through DO are capable of withstanding the biomechanic requirements associated with implant loading [17-18]. These outcomes are similar to those seen when implants are inserted into non reconstructed alveolar bone.

Although autogenous bone procurement is required for vertical GBR, which lengthens recovery periods and raises postoperative complications, the approach is an effective reconstructive method [21-22]. Furthermore, early membrane invasion may result in infection, which could harm the rehabilitation's overall success. This method has primarily been used for small abnormalities with estimated vertical bone loss of 2 to 7 mm. Our work and other articles support the reliability of DO as an approach for bone augmentation [23-24]. In excess of 15 mm of vertical bone augmentation can be achieved and does not require bone grafting, which lowers morbidity [25-26]. Neo-histogenesis, an on-going expansion of the adjacent tissue, may provide an additional benefit. The danger of bone exposing and wound breakdown is notably low. Infection frequency was 0% in both this investigation and other publications [20-26]. Without a stable base, the bone transplants in the defect area may shift as a result of local stress, resulting in the collapse of the bone-augmented area and an unintended outcome [21-22]. Therefore, to guarantee excellent biocompatibility, the barrier membranes in the GBR technique must have sufficient retention potential, supportability, and stiffness [17-18]. While they may isolate cells selectively, conventional barrier membranes are not as flexible and have difficulty provide enough retention and protection for the locations where bone regeneration occurs. Non-absorbable expanded poly-tetra-fluor-ethylene membranes (ePTFE) and absorbable collagen are two types of these membranes [12-13].

The traditional barrier membranes are difficult to install over large bone defects and easily create micro-motion that inhibits blood flow, making it difficult to maintain an optimal and stable area for bone regeneration [14, 17]. When applied to alveolar bone with large vertical or horizontal bone defects, titanium mesh exhibits outstanding osteo-genic performance and great mechanical properties, according to several clinical studies [18-20]. Alveolar DO is an additional method for restoring vertically degraded alveolar ridges. Although maxillofacial anomalies have been treated with it recently, it was originally intended to treat orthopaedic disorders. It has been suggested to correct the edentulous alveolar ridges' vertical inadequacies [21-22].

Data suggest that DO may provide a better prolonged prognosis than GBR in terms of maintaining bone growth and peri-implant resorption of bone during prosthetic loading. Implant success rates vary considerably between the DO versus GBR groups, but implant rates of longevity differ [16-17,23- 24]. It is important to note that while GBR allowed synchronous correction of a vertical bone defects as well as a horizontal bone defects, the distraction device utilised in the present investigation only allowed rectification of the vertical defect [18-21]. When dealing with miniature faults or an amalgamation of horizontal as well as vertical defects, GBR approaches may be more appropriate [21- 22]. There is reduced space for osteotomies and because of the size of the distraction device, DO utilising intraoral extra-osseous distractors in a single-tooth spacing may actually be more challenging to complete [16-17].

## Conclusion:

Data shows that distraction osteo-genesis is more predictable for long-term prognosis of vertical bone growth. However, all approaches help to repair vertically resorbed edentulous ridges.

## Figures and Tables

**Table 1:** Values of bone resorption at different time durations in different bone augmentation techniques

	**GTR +Titanium mes**h	**GTR + e-PTFE membranes**	**DO**	**Onlay bone grafts**
BRIP				
Mean± SD(mm)	1.46±0.7	1.57 ± 0.4	0.48±0.3	0.67±0.2
P value	0.001*			
BRAC				
Mean± SD(mm)	1.38±0.9	1.47±0.9	0.61± 0.5	0.76±0.7
P value	0.011*			
BD 1				
Mean± SD(mm)	1.94±1.2	1.97± 1.4	1.24 ±0.6	1.35±0.4
P value	0.03*			
BD 2				
Mean± SD(mm)	1.99 ±1.1	2.05± 1.4	1.35±0.6	1.46± 0.3
P value	0.002			
BR3				
Mean± SD(mm)	2.17 ± 1.1	2.26±0.12	1.52±0.4	1.67±0.52
P value	0.001*			
TBD 3				
Mean± SD(mm)	3.63±1.3	3.83±1.8	2.00±0.8	2.34±0.32
P value	0.021*			
*indicates statistically significant difference.

**Table 2:** Implant survival rate and success rate at different follow ups

	**Placement to loading**	**Loading to 1 year**	**1-2 years**	**2-3 years**
GBR ± Titanium mesh membranes				
Cumulative survival rate (%)	100	100	100	100
Cumulative success rate (%)	100	84.4	84.4	76
GBR ± ePTTE membranes				
Cumulative survival rate (%)	100	100	100	100
Cumulative success rate (%)	100	82.3	82.3	74
**DO**				
Cumulative survival rate (%)	100	100	100	100
Cumulative success rate (%)	100	100	100	95.2
Onlay bone grafts				
Cumulative survival rate (%)	100	100	100	100
Cumulative success rate (%)	100	100	97.4	93.2
